# Impact of Magnetization on the Evaluation of Reinforced Concrete Structures Using DC Magnetic Methods

**DOI:** 10.3390/ma15030857

**Published:** 2022-01-23

**Authors:** Paweł Karol Frankowski, Tomasz Chady

**Affiliations:** 1 Doctoral School, West Pomeranian University of Technology in Szczecin, Piastow St. 19, 70-310 Szczecin, Poland; 2 Faculty of Electrical Engineering, West Pomeranian University of Technology in Szczecin, ul. Sikorskigo 37, 70-313 Szczecin, Poland

**Keywords:** nondestructive testing NDT, nondestructive evaluation NDE, magneto-optical (MO) sensor, anisotropic magneto resistance (AMR) sensor, reinforcement bars detection, rebars, concrete inspection, reinforced concrete

## Abstract

The magnetic method is the most promising method that can be used to inspect large areas of reinforced concrete (RC) structures. Magnetization is a crucial process in this method. The paper aims to present the impact of the magnetization method on the results in the detection of reinforced bars (rebars) and the evaluation of concrete cover thickness in reinforced concrete (RC) structures. Three cases (without magnetization, same pole magnetization, and opposite pole magnetization) were considered in the experiments. Results achieved in all the methods are presented and evaluated. Two different sensing elements were used in the measurements: a magneto-optical (MO) sensor and an AMR sensor. The advantages and disadvantages of both mentioned transducers are presented and discussed in the context of a large areas inspection. The new approach involves using various magnetization methods to improve measurement results for complex structures.

## 1. Introduction

### 1.1. Nondestructive Methods of Testing Concrete Structures

For over a century, reinforced concrete (RC) has been a dominant construction material for structures of every type and size. Usually, buildings of this kind are designed for 50–100 years of operating time. However, the remaining lifetime of a specific structure is challenging to estimate because many different factors have influences. Many structures built at the beginning of the twentieth century are still in service [1,2]. Therefore, in most countries, periodic inspections of old structures are required by a building code (usually once per five years). Even new construction acceptance tests are conducted to determine if the requirements of a specification or contract are met. The requirements may involve verification of the class, diameter, and arrangement of the rebars in the concrete.

Reinforced concrete could be tested in many different ways. The methods range from destructive, through semi-destructive (where the concrete is partially damaged), to utterly nondestructive testing (NDT). The NDT methods are usually cheaper and faster than methods of other groups. Unlike the destructive and semi-destructive, they can also be easily used in many points of the tested object. Therefore, they better reflect the actual state of the facility.

**Figure 1:**
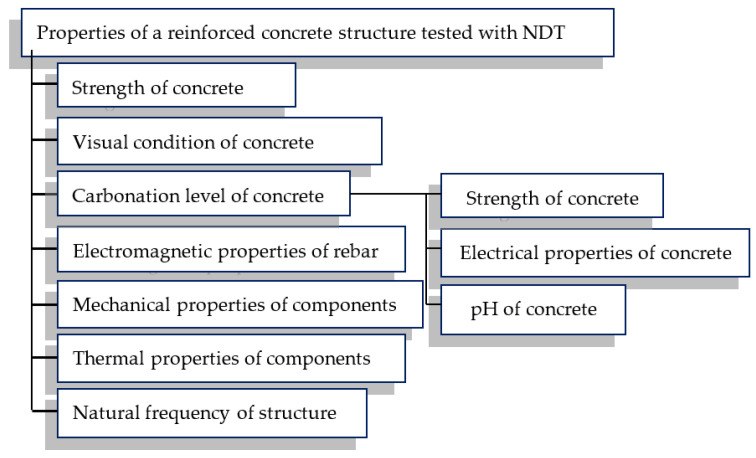
Properties of reinforced concrete structures that can be examined by NDT methods.

A full review of NDT methods used in construction diagnostics, along with their advantages and disadvantages, is given in [3]. The properties of a reinforced concrete structure which can be examined with NDT methods are presented in Figure 1.

As described in [3] and presented in Figure 1, most of the NDT methods used in civil engineering are designed to evaluate concrete. Only methods that use an electromagnetic and mechanical wave can be effectively used for direct reinforcement assessment. The following methods can be distinguished in the mechanical group: high-frequency, active ultrasonic testing methods [3,4,5]; low-frequency-active mechanical methods [3,6], and passive-acoustic emission (AE) [7].

Electromagnetic methods are not universal, but on the other hand, they have many advantages over mechanical methods. The most crucial difference is that the results of the mechanical methods are affected by many factors because various phenomena may disturb the propagation of mechanical waves in complex structures. Therefore, electromagnetic and magnetic methods are preferred to assess reinforcement elements in concrete structures.

The electromagnetic methods may be used to localize rebars in the structure, precisely estimate basic structure parameters (such as the thickness of the concrete cover, the rebar’s diameter, the rebars class [8,9,10], and detect corrosion or other flaws [11,12,13]). The most significant advantages of the methods from this group are the direct impact on reinforcement, the low damping of electromagnetic waves by concrete and the high spectrum of frequencies that can be used.

**Figure 2:**
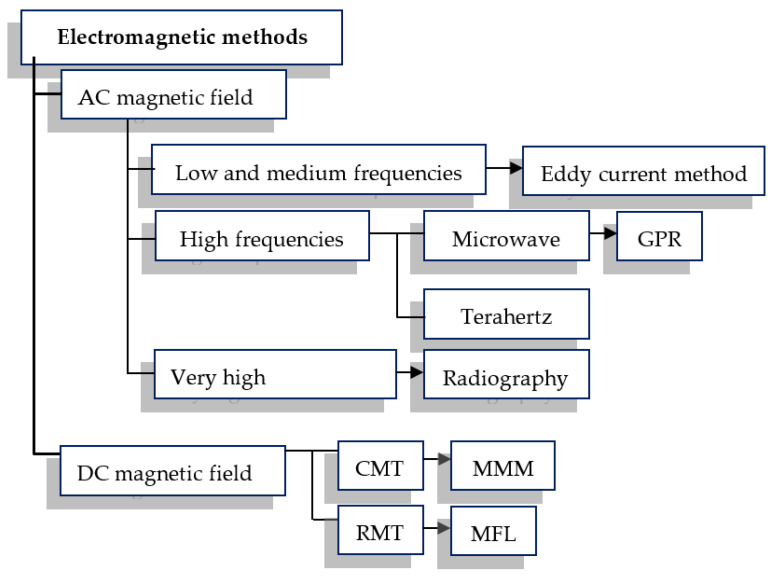
Classification of electromagnetic NDT methods according to the excitation frequency.

NDT electromagnetic methods can be categorized by the utilized excitation frequency (Figure 2). This frequency is crucial for all methods that use mechanical or electromagnetic waves. It affects resolution and an effective range. The same method may have good resolution and limited range (high frequency) or good effective range and low resolution (low frequency). In simplification, it can be assumed that the smallest size of the defect that can be detected is approximately comparable to the excitation frequency wavelength [14]. The penetration range depends on the frequency of excitation and magnetic permeability of concrete and steel. The fundamental division of NDT methods due to the frequency of excitation is shown in Figure 2.

The most important AC magnetic field NDT method used in civil engineering is the eddy current (EC) method. In this method, the typical excitation frequency range is from 0.5 to 10 kHz (for reinforced concrete structures). The eddy current method can be used not only to detect the presence of rebars but also to determine the thickness of the concrete cover, the rebar’s diameter, the alloy of reinforcing bars (due to different electrical properties), or even to detect corrosion of rebars [8,9,10,11,12,13]. The effective range of the eddy current method is from 0 to about 100 mm. Results can be really accurate and relatively easy to interpret. Lower excitation frequencies may be used in some versions of the magnetic flux leakage (MFL) and the magnetic force induced vibration evaluation MFIVE method [3] or in the method similar to MFIVE described in [6]. Both of these methods use low-frequency magnetic waves to induce rebar vibration. Natural frequencies of the reinforcement can be used to detect structure debonding, which is usually caused by corrosion.

Another important electromagnetic method is ground-penetrating radar (GPR). The standard operating frequency ranges from 100 MHz to 3 GHz. Rebars can be detected from the distance of several centimeters up to ten meters or more (when other electromagnetic methods have the maximum detection range not bigger than 200 mm). However, results are difficult to interpret and not very accurate [15,16]. The terahertz technique is rarely used due to the limited penetration in concrete, which is usually characterized by high water content, strongly damping electromagnetic waves at these frequencies. Higher frequencies are used in radiography, which can be very effective but, on the other hand, possess many limitations. The source and detector usually must be placed on both sides of the object. Moreover, this method generates risks for human health [3].

Inspection methods utilizing DC magnetic field can be divided into two categories: continuous magnetization techniques (CMT), also called active magnetic inspection (AMI) and residual magnetization techniques (RMT), called passive magnetic inspection (PMI). In the case of CMT, not only receiving devices but also excitation is required.

The leading representative of CMT is the magnetic flux leakage method (MFL). The method is commonly used in the inspection of ferromagnetic parts and components. However, currently, the adaptations of this method for civil engineering are also popular.

In the MFL method, the detector is usually placed between the poles of the magnets or electromagnet) to detect the leakage field. The relative permeability of concrete, stones, water, and the air is close to 1. Therefore they have practically no influence on the magnetic field distribution. The reinforced bars (rebars) made of steel as ferromagnetic materials concentrate the magnetic flux. In this way, the magnetic field is influenced by rebars and can be used to localize them in the concrete structure. The magnetic flux can be disturbed by discontinuities in the material, such as breaks or cracks [12]. The magnetic flux leakage caused by rebar inhomogeneity can be detected at a distance in the range of the typical concrete cover [17,18].

In some cases, the MFL method can be used to determine the material loss caused by corrosion [19,20,21,22]. Magnetic methods also allow to identify rebar diameter [23]. The magnetic flux leakage method can also be used for structural health monitoring [22]. Other active magnetic methods, such as Barkhausen emission (MBE), magnetoacoustic emission (MAE), stress-induced magnetic anisotropy (SMA), or magnetic powder method, usually are not used for the evaluation of reinforced concrete structures. The magnetic field is higher in the case of the active magnetic methods (CMT). However, the CMT methods also have disadvantages like longer measurement time, equipment deployment, and power consumption [3].

Residual magnetization methods are more economical and straightforward. The basic RMT is the magnetic memory method (MMM). The method can be used to detect abnormal conditions arising from changes in crystalline structures resulting from stress concentration, corrosion, or cracks. One of many versions of MMM is iCAMM (infrastructure corrosion assessment magnetic method). This method works through passive magnetic inspection under the effect of the Earth’s magnetic field.

### 1.2. Novelty and Significance of the Research

Periodic evaluation of reinforced concrete structures is required by national law in most countries. However, in many cases, such inspection can be problematic. Standard ‘in point’ tests can be misleading (most of the structure is not checked). The point-to-point scans also cannot be used in large areas because tests of this kind are usually very time-consuming. The obvious solution is to use area tests. In such a way, the investigation time is significantly reduced and received results are reliable. However, currently, there is not even one method that can be used in that way on a large scale. Area tests potentially can also be used as a pilot or preliminary evaluation before applying other more precise methods. There are only a few methods that theoretically can be used for such evaluation. This group includes primarily visual testing, radiography, and thermography. Unfortunately, these methods have many limitations (e.g., thermography can be used only if the concrete cover is low [8,24]; radiography requires specialized equipment, generates risks for human health, and elements of the system must be placed on both sides of the object) and they are often insufficient. The full summary of the area testing methods is shown in [3]. The magnetic methods are not always considered to be good for area tests. However, this method possesses many advantages over others tests mentioned before. Tests executed with the magnetic method are cheap, the principle of operation is easy to understand and use, the used magnetic wave can avoid damping caused by concrete cover. The test showed that the magnetization method is crucial for the effectiveness of this method. The potential of the active and passive magnetic methods is presented in further sections of the paper.

### 1.3. The Article Outline

In the introduction of this paper, first, the importance of nondestructive testing (NDT) in periodic tests of reinforced concrete structures has been described. A brief overview of the NDT methods used in the construction sector is also presented. Next, the significance of the conducted research was indicated.

The Section 2 (Materials and methods) presents the tested samples and measurement systems. The section has much attention to magneto-optical (MO) sensors. The MO elements, one of the few magnetic field detectors, are designed for area testing. The evaluation of ferromagnetic objects remote from the sensor as much as in the case of reinforced concrete is an unusual issue for this type of sensor, which is intended and designed for surface testing. Therefore, before the tests, their accuracy in the case of reinforced concrete structure was doubtful. For more detailed investigations, AMR sensors connected in one matrix were proposed. In this section, examples of received results and algorithms of data processing are discussed.

The results of the measurements were placed in the Section 3. First, the entire section is briefly described. Next, results received for the MO sensor are presented. The experiments with the MO sensor show both the influence of magnetization on increasing the ability to detect rebars and the application potential of the MO-sensors.

In the other subsection, results received for three different samples and three different magnetization variants are presented. All experiments were conducted with the AMR sensor. The main point of the subsection is to show how significant the impact of the magnetization method on received results can be. The impact is even stronger for more complex samples. This part also presents the disadvantages of the passive method, which also becomes more significant during the tests on more complex samples.

The obtained results are summarized in the Section 4 In particular, the magnetization aspect is discussed in this part. The section ‘Conclusions’ discussed whether the magnetic method is finally suited for area testing and how the tests of this kind fall on the background of other methods. The two tested sensors are also compared in this part. The advantages and disadvantages of both systems are presented, and applications of the sensors have been proposed. In the section also plans for further research on the magnetic method for area testing are presented.

## 2. Materials and Methods

### 2.1. Measuring Systems and Samples

#### 2.1.1. Test Samples

**Figure 3:**
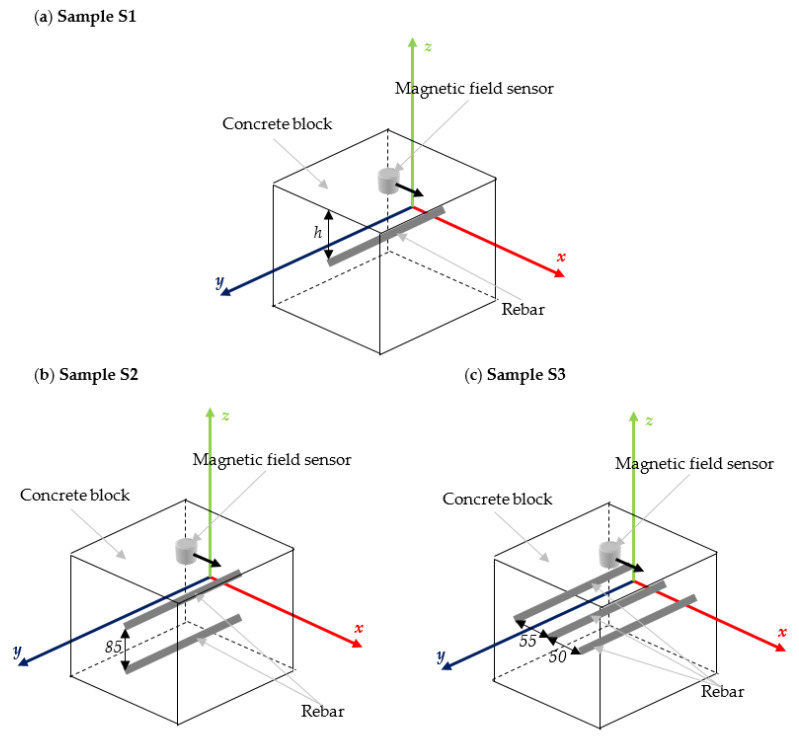
The samples used in the experiments; (**a**) sample S1 (with single rebar); (**b**) sample S2 (with two rebars one under the other); (**c**) sample S3 (with three rebars next to each other).

The main aim of the article is to investigate the influence of magnetization on the effectiveness of magnetic nondestructive testing methods in the evaluation of reinforced concrete structures. For this purpose, the three different samples are examined: S1—the sample with single rebar (Figure 3a); S2—the sample with two rebars, one placed 85 mm under the other (Figure 3b), and S3—the sample contains three rebars, all rebars placed one next to each other (Figure 3c). In the third sample, distances between rebars are 55 and 50 mm. The magnetic sensor was moved above the sample in a line perpendicular to the reinforcement bars. The distance between the rebar and the sensor (thickness of the concrete cover) is marked as *h* (Figure 3a). The results were obtained using an integrated AMR transducer that allows measuring three field components.

Configurations of the samples are presented in Figure 3. The magnetic transducer was moved along the *x*-axis, while rebars were positioned along the *y*-axis.

#### 2.1.2. Systems for Active Magnetic Inspection

**Figure 4:**
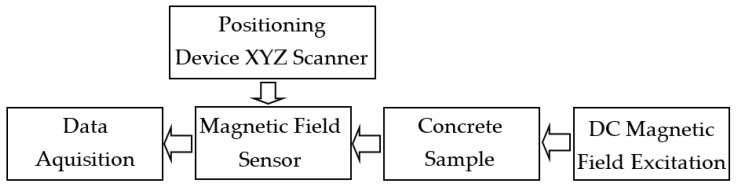
Block scheme of the measuring system.

The measuring system consisted of four subsystems: excitation subsystem, positioning subsystem, magnetic field transducer, and data acquisition subsystem. The general block scheme of the system is presented in Figure 4. All subsystems are described in the following sections.

**Figure 5:**
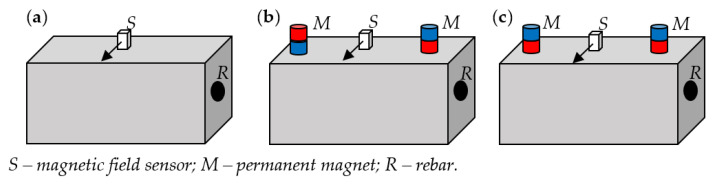
System configurations used in the experiments; (**a**) reference configuration; (**b**) configuration of opposite poles magnetization (OPM); (**c**) configuration of same poles magnetization (SPM).

The simplest solution to magnetize reinforcement bars (rebars) can be achieved using permanent magnets. In the presented systems, two neodymium magnets in two different configurations were used for this purpose. The reference configuration was without any magnets, as shown in Figure 5a. In the second configuration, magnets have opposite poles facing the sample (Figure 5b). In the third configuration, the magnets were directed to the sample with the same poles (Figure 5c). The magnets were placed on both sides of the sensor at a distance of 500 mm.

**Figure 6:**
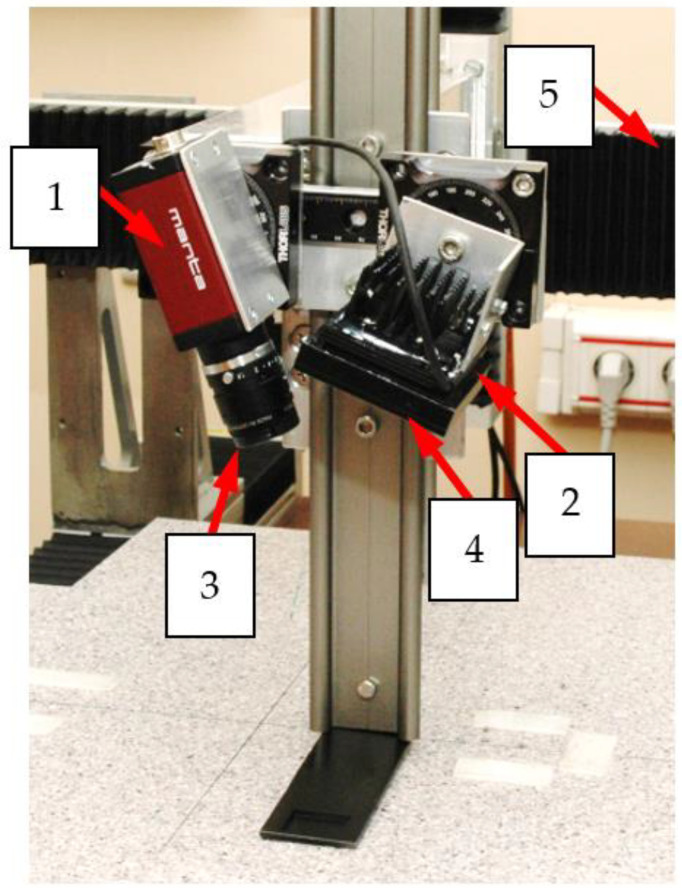
Photo of the MO transducer attached to the XYZ scanner: 1—camera; 2—the source of monochromatic light; 3 and 4—linear polarizers; 5—XYZ scanner.

In the experiments, a two-dimensional area over the sample surface was scanned. The area directly above the rebars is tested with a positioning system. The example of positioning subsystem is shown in Figure 6.

The magnetic field sensor is an essential part of the system. Magnetoresistive (MR) and Hall effect sensors are of the greatest industrial importance among the magnetic field sensors. The Hall effect components account for approximately 85% of the world’s production of magnetic sensors for DC and low-frequency applications. The MR sensors account for around 10%, and their market share grows [25].

The most used MR sensors are anisotropic magnetoresistors (AMR) and giant magnetoresistive effects (GMR) elements. The AMR and GMR sensors have high sensitivity and field resolution. Elements of this kind can operate even in the pT range. However, they can be permanently affected by strong magnetic fields and GMR sensors have a high hysteresis.

The Hall effect sensors have several advantages over MR elements. They show no saturation effects and can measure strong magnetic fields. For these reasons, the Hall effect sensors are preferably used at magnetic fields higher than 1 mT. They are the first choice in many industrial applications. However, large offset and relatively low sensitivity limit both the accuracy of the measurements and the minimum value of the magnetic field that can be measured. One of the issues examined in this research is testing non-magnetized reinforced concrete structures using magnetic methods. The MR sensors seem to be much better suitable for this purpose.

Most magnetic field sensors can measure the magnetic field at one point. The exception is magneto-optical (MO) sensors, which are well suited to constructing an area testing system. Therefore, magneto-optical (MO) sensors are preferable for testing large-scale reinforced concrete structures. The Faraday magneto-optical effect is used in MO sensors [26,27]. The main advantage of this solution is the immediate obtaining of the 2D field distribution over the sample surface.

#### 2.1.3. Measuring System with Magneto-Optical Sensor

**Figure 7:**
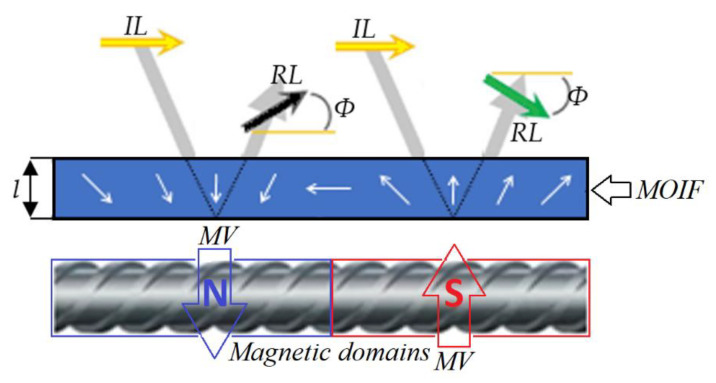
Operating principle of the MOIF. MOIF—Magneto-Optical Indicator Film (sensor); MV—Magnetic Vector; IL—Polarization Plane of Incident Light; RL—Polarization Plane of Reflected Light; *Φ*—Angle of Faraday rotation.

The Faraday magneto-optical effect is used in MO sensors. This effect describes an interaction between light and a magnetic field in a medium. The plane of polarization of linearly polarized light rotates parallel to the propagation direction of light waves passing through the magneto-optical medium. The mechanism of the Faraday effect is explained in Figure 7 [26,27].

**Figure 8:**
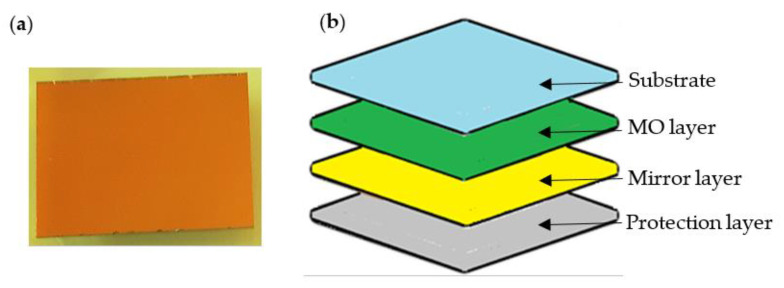
Magneto-optical sensor; (**a**) the photo of the A-type MO sensor in the protective packaging. (**b**) schematic showing the functional layers of the Magneto-Optical Indicator Film (sensor).

The MO sensor presented in Figure 8a consists of four layers, as shown in Figure 8b. Additional layers are necessary to improve the quality of the measurements. The mirror layer (for visible spectral range) is used to improve the sensor reflectivity. For mirror protection, the resistant material layer is used. The sensor also contains anti-reflection coated glass [26,27].

**Figure 9:**
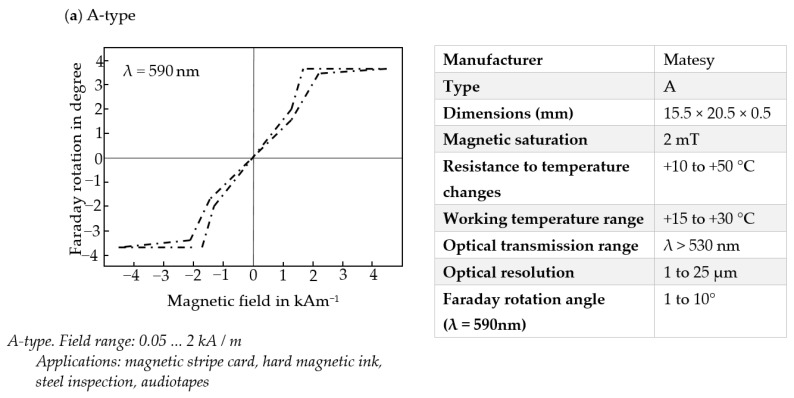

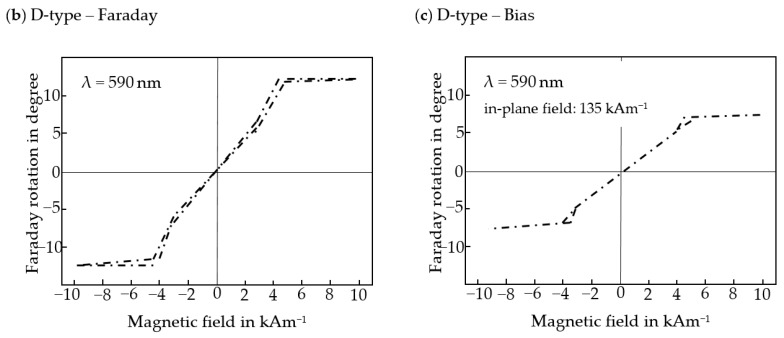
Parameters and approximate curves of characteristics-utilized MO-sensor; (**a**) A-type sensor: plot of magnetic field vs. Faraday rotation *Φ* (λ = 590 nm), and selected parameters of the sensor; (**b**) D-type Faraday version of the sensor: plot of magnetic field vs. Faraday rotation *Φ* (λ = 590 nm); (**c**) D-type bias version of the sensor: plot of magnetic field vs. Faraday rotation *Φ* (λ = 590 nm). (Based on materials received from the manufacturer Matesy).

The most important advantage of the MO-sensor over other magnetic field sensors is the large area of observation of the magnetic field and the relatively high resolution. The most significant advantage of MO-sensor over other magnetic field sensors is the large area of magnetic field observation and relatively high resolution. The manufacturers offer sensors with diameters up to 3 inches. A few different types of MO transducers are used in many different applications [26,28]. Parameters and characteristics of the type A sensor used in the experiment are provided in Figure 9. The sensitivity of this MO-sensor is comparable to the Hall effect elements.

The type A sensor used in the experiments is an out-of-plane (OOP). The MO-sensors of this kind are generally more sensitive but have a smaller range and nonlinear characteristics. The hysteresis (Figure 9a) can also cause difficulties during measurements (in the case of less sensitive MO-sensors, there are no such problems). The A-type is chosen because of the lowest dynamic range (significant visible changes with minor magnetic field changes). An alternative to the A-type transducer in this kind of application is a D-type transducer. Sensors of this kind are more sensitive than A-type; field range is from 0.03 to 5 kA/m and can be used to test printed magnetic inks or steels alloys. The sensors are sensitive, but it also depends on the quality of the camera and other elements. There is another valuable property of the D-type element.

The D-type element can be working in two modes:

- Faraday: for applications without external excitation;
- Bias: for work in the environment of an external magnetic field. In this mode, performance is weaker, but other types of sensors would lose their performance entirely. This mode is used mainly with magnetically very soft materials, like inks.

**Figure 10:**
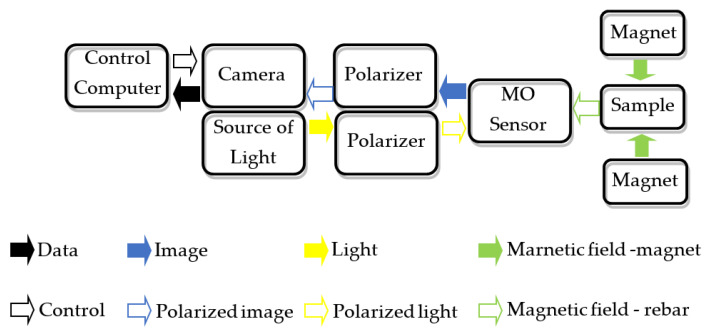
Block diagram of the system with the MO-sensor.

The MO-transducers require a relatively complex setup. The block diagram of the system with MO-transducer is shown in Figure 10, and the setup photo is presented in Figure 6.

#### 2.1.4. Measuring System with a Magnetoresistive Sensor

Systems based on MR sensors are less complex than these based on MO sensors. Moreover, the AMR sensors with three sensitivity axes are better suited for more accurate investigations of reinforced concrete structures.

AMR (anisotropic magneto resistance) elements belong to the MR group of sensors. The resistance of these elements decreases when a magnetic field is applied. This function is dependent on the direction of the magnetic force lines applied to the element (anisotropic). The material of the AMR element is an alloy of nickel, iron, and other metals (ferromagnetic). In these experiments, integrated transducer HMC5883L was used. The sensor has few advantages over GMR. The sensitivity is high, much higher than in the case of the MO sensor. Nevertheless, lower than it could be in the case of GMR [29].

On the other hand, the sensitivity of GMR would be too high for this application. With the use of ‘reset strap drive’ the internal offset of the sensor and its temperature dependence is corrected for all measurements. This option could be helpful in the vicinity of large magnetic fields. In opposite to GMR, AMR sensors clearly indicate the results of the magnetic field direction. Because the positive and negative sides have symmetric characteristics, the same operation is performed even if the north and south poles of the magnet are reversed. This characteristic is used to improve the reliability and accuracy of the data. The sensor also has high linearity and low hysteresis.

### 2.2. Methods of Processing the Results

#### 2.2.1. Measurement Results Processing in the System with MO Sensor

**Figure 11:**
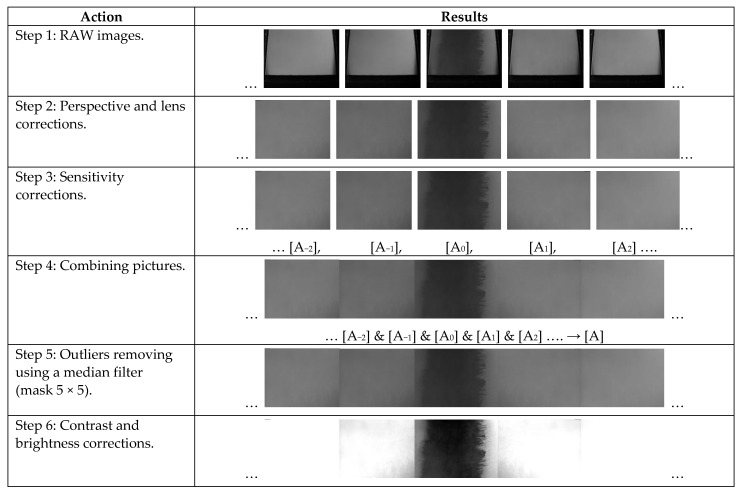
The processing of images obtained with the MO sensor; same pole magnetization (SPM); Sample S1; *h* = 0.5 mm.

The results obtained from MO systems usually do not require complicated processing and are available in real-time. Nevertheless, in some cases, such as a high thickness of concrete cover *h*, even minor image changes have to be detected. Therefore the following algorithm of the image enhancement was implemented. First, the algorithm extracts the active area of the MO sensor from the image obtained from the camera. Then, since the axis of the camera lens was not perpendicular to the sensor surface, it was necessary to correct the perspective. The next step is to reduce the geometric distortions caused by the lens. Images processed in this way are saved in the system memory. Due to the relatively small size of the sensor area, the final image [A] consisted of several (5 to 7) images [A_n_] taken at subsequent positions above the sample. The sensitivity of the MO transducer is not the same at different places on the sensor surface. Therefore, the images [A_i_] are corrected using a coefficients matrix calculated from a uniform DC magnetic field measurement. In the cases of small (0–20 mm) or big (80–100 mm) thickness of concrete cover (*h*), it is also necessary to correct the non-linearity of the characteristic and hysteresis presented in Figure 8a. In order to remove noises, a 2D-median filter with a 5 × 5 mask is applied to the image [A]. In the last step, contrast and brightness were corrected. Effects of the processing are shown in Figure 11.

The MO sensors enable testing areas of objects under investigation without time-consuming point-by-point scans. Unfortunately, sensitivity, linearity, and repeatability are limited. Moreover, the images are noisy. The problems only to some degree, can be caused by hardware limitations (polarizers or video cameras). The MO sensors could be a solution for a preliminary evaluation.

#### 2.2.2. Measurement Results Processing in the System with MR Sensor

**Figure 12:**
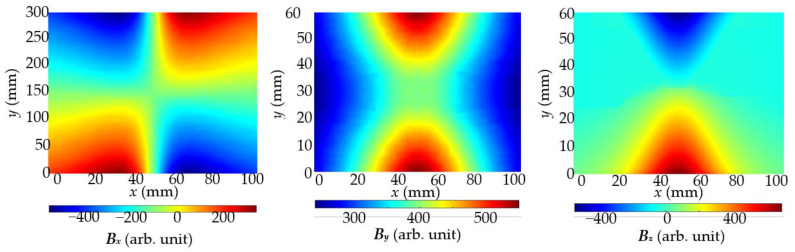
Magnetic field *B_x_*, *B_y_*, and *B_z_* components measured in case of the same pole polarization and sample S1 with single rebar. Measurements were carried out with the AMR sensor; concrete cover thickness *h* = 20 mm.

MR systems are much more sensitive than MO systems. Moreover, systems of this kind can deliver information about three components of the magnetic field. In further investigation, measurements were taken by moving the transducer with a 1 mm step in the *x*-axis and 10 mm in the *y*-axis direction. The measurements were very time-consuming. Examples of results received using opposite poles polarization for inspection of the sample S1 are presented in Figure 12.

## 3. Results

The results of the experiment are discussed in two subsections. The first part presents the magnetic field distribution measurements using MO-sensor and their application for rebars detection. The experiments can be assumed as preliminary studies. The measurements with the MO-sensor are carried out quickly, and they are easy to interpret. However, the sensitivity of the MO-sensors is lower than the AMR sensor, and there is no possibility to measure *x*, *y*, and *z* induction components. In this case, only sample S1 with single rebar is tested (all samples were tested with the AMR sensor as shown in the following subsection). The experiments with the MO sensor show both the influence of magnetization on increasing the ability to detect rebars and the application potential of the MO-sensors. The same pole magnetization (SPM) is used in this experiment.

In the following subsection, results received for three different samples and three different magnetization variants are presented. All experiments were conducted with the same magnets (having different orientations against the rebars). Therefore, the magnetization effect is weaker for samples with a bigger concrete cover thickness. In addition, always the same single AMR sensor was used. The main point of the experiments is to show the impact of the magnetization method on received results. Tests prove that the impact is even more significant for more complex samples. Experiments carried out on the samples simulating reinforced mesh (samples S2 and S3) showed that the CMT (Continuous magnetization techniques) were much more effective than RMT (residual magnetization techniques). Moreover, the SPM (same pole magnetization) allows identifying rebars more straightforwardly than OPM (opposite pole magnetization).

### 3.1. Experiments with the MO-Transducer

Experiments using the MO-sensor for sample S1 (with single rebar) were conducted to show the differences between CMT and RMT. Rebars are magnetized every time up to the same level and in the same orientation. The SPM was selected as a method of magnetization. As a reference, the same experiment was also conducted with the non-magnetized rebar. Experiments were taken with the step of 5 mm along the axis *z* (change of concrete cover thickness *h*), and 20.5 mm along the axis *x* (size of the sensor is 15.5 × 20.5). In this way, continuous measurements were obtained without any gaps. The thickness of the concrete cover *h* was changed in the range from 0.5 to 100 mm.

**Figure 13:**
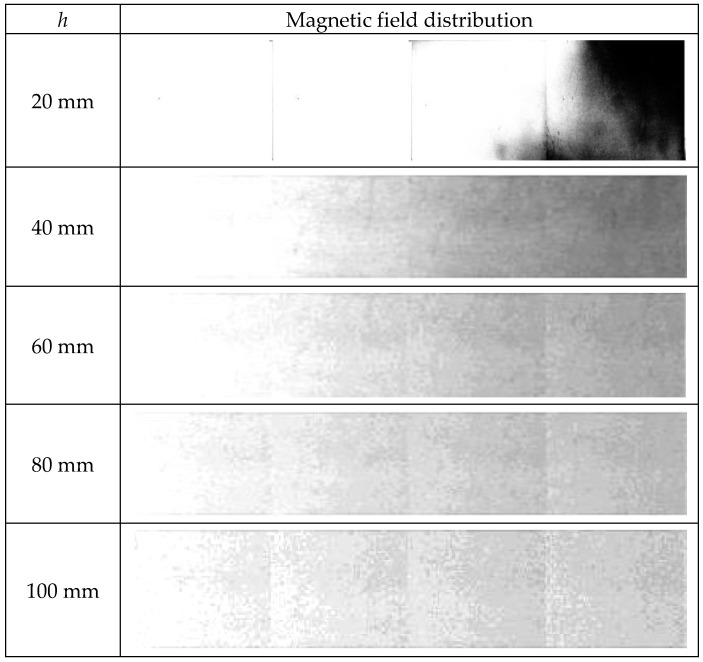
Magnetic field distribution measured with the MO-sensor for different concrete cover thicknesses; same pole magnetization; only half of the measurements are shown.

Predictably, experiments have shown that magnetized rebar can be detected with a much greater concrete cover than a non-magnetized. When the non-magnetized rebar is challenging to detect with a cover thickness above *h* = 20 mm, the magnetized rebar could be detected from a distance of more than 100 mm. However, the readability of the graphs for large cover thicknesses is limited. Examples of the measurements received for thick concrete cover are shown in Figure 13. Only half of the measurement results are shown (the other half is symmetrical).

**Figure 14:**
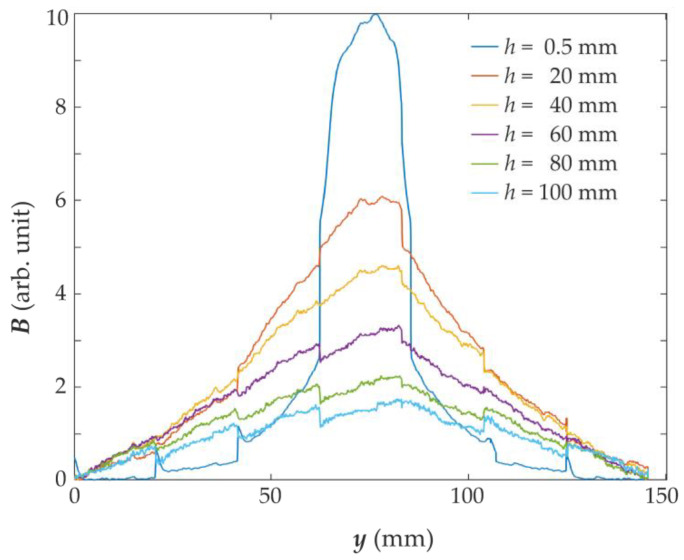
Impact of concrete cover thickness on the MO-sensor measurements. The average line profile of the magnetic field was measured using MO-sensor with the same pole magnetization SPM by moving the sensor along the *y*-axis.

Plots showing the magnetic field distribution over the magnetized rebar vary depending on the thickness *h* of the concrete cover. Examples of such characteristics are shown in Figure 14. They are repeatable and unambiguous. Therefore, on their basis, it is possible to estimate the location of the rebar, the thickness of the concrete cover, and possibly other parameters of the structure, as was the case in [9,10].

In existing civil engineering constructions, the thickness of the concrete cover is usually between 10 mm to 50 mm over the reinforcing bars. When the reinforcing bars are not magnetized, the MO sensors are not sensitive enough to detect rebars from such distances. However, when the bars are magnetized, the efficiency of the MO sensors is sufficient. Thus, sensors of this type are suitable for the CMT and not for RMT. Examples of calculated signal to noise ratio (SNR) values are presented in Table 1.

The SNR was defined as:

$$\mathrm{SNR}=20\log\frac{A_{\mathrm{signal}}}{A_{\mathrm{noise}}}$$

### 3.2. Influence of Rebars Magnetization Method on Magnetic Field Distribution

All experiments in this section were taken with the step of 5 mm along the axis *z* (change of concrete cover thickness *h*), from 20 to 70 mm (typical concrete cover thickness).

The step along the axis *y* was equal to 20 mm and experiments were taken from −100 to 100 mm. Position 0 is a position in the middle of the rebar.

The step along the axis *x* was equal to 2 mm and experiments were taken from 0 to 98 mm. Rebars in S1 and S2 are placed in position 27 mm (axis *x*). In the case of S3, the middle rebar is placed in this position.

Magnets were moving together with the sensor and were placed on both sides of the sensor at a distance of 500 mm.

**Figure 15:**
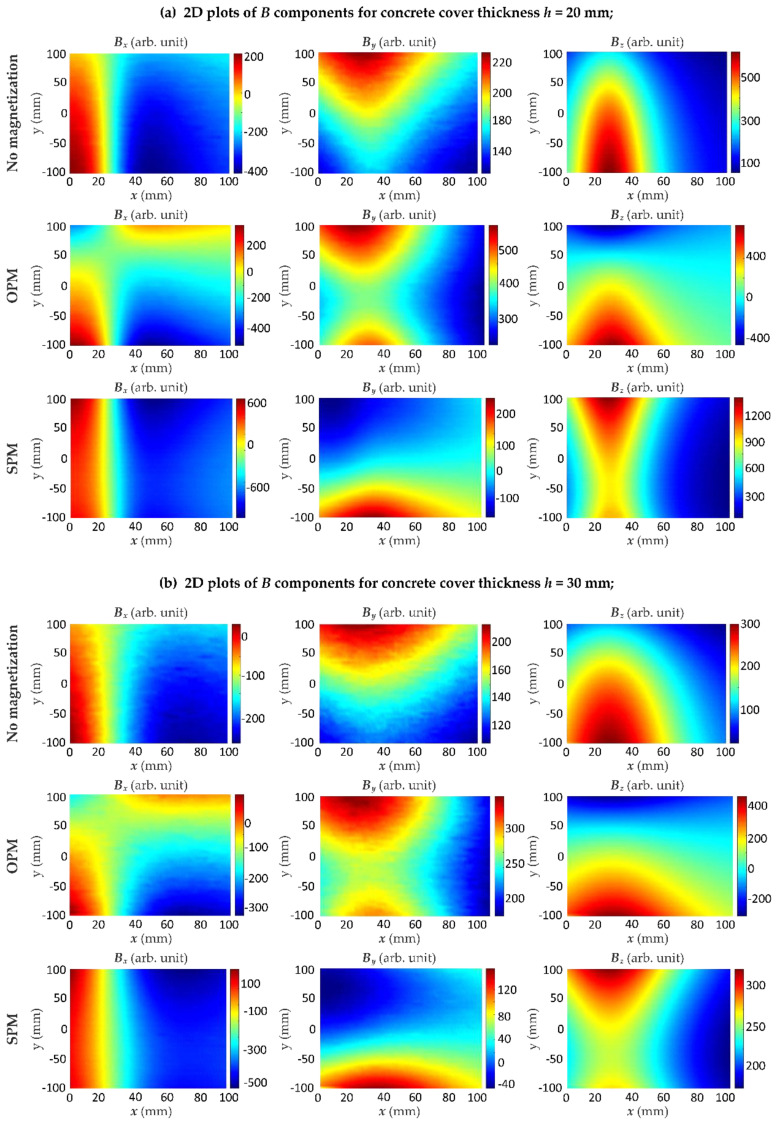

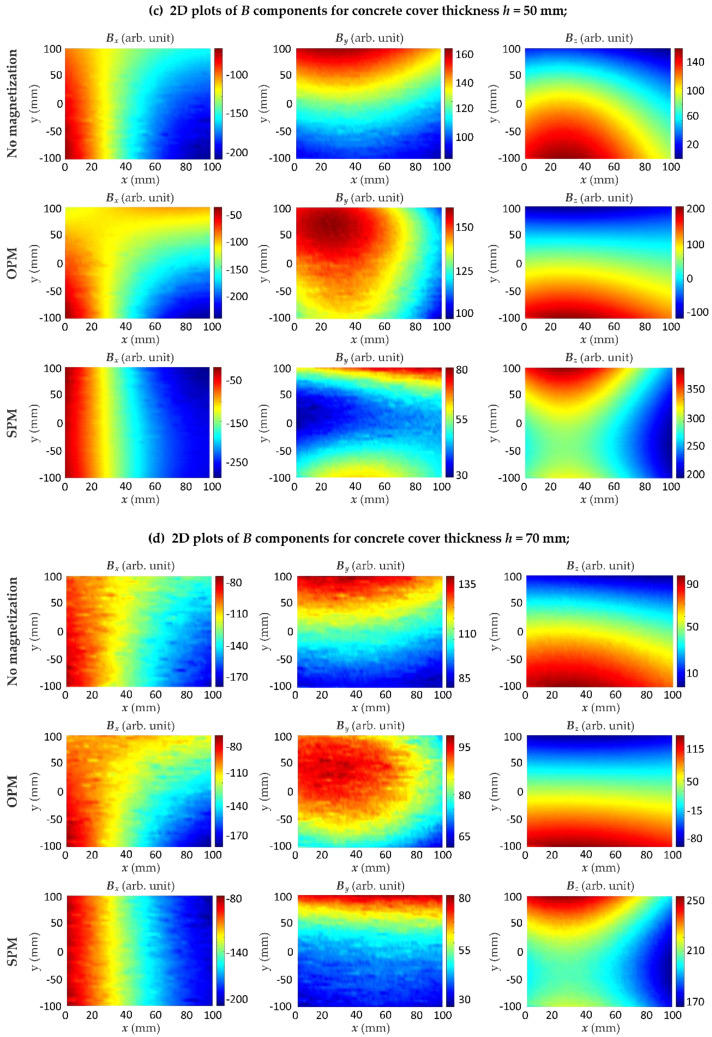
Results of 2D measurements using the AMR-sensor obtained for different variants of magnetization and different thicknesses *h* of concrete cover; experiment conducted for the sample S1 with single rebar; SPM—same pole magnetization, OPM—opposite pole magnetization; concrete cover thickness: (**a**) *h* = 20 mm; (**b**) *h* = 30 mm; (**c**) *h* = 50 mm; (**d**) *h* = 70 mm.

**Figure 16:**
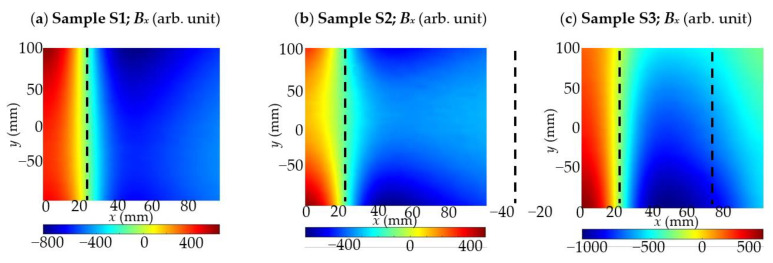
Selected results of 2D measurements of magnetic field component *B_x_* with the depicted position of the rebar (dashed line); (**a**) sample S1—single rebar; (**b**) sample S2—two rebars one under the other; (**c**) sample S3—three rebars next to each other.

In the first set of experiments, the measurements were carried out for the sample S1 using different magnetization methods. The results for different thicknesses *h* of concrete cover are presented in Figure 15. The second set of experiments was carried out with three different samples S1, S2 and S3, shown in Figure 3. The measurement results were symmetrical concerning the rebar, and therefore the measuring range has been reduced nearly by half. Positions of the rebars were depicted on the plots in Figure 16 by dashed lines.

Figure 15 shows that the influence of magnets on the rebar decreases when the cover thickness *h* is increasing, and thus, the magnetic field measured by the sensor also decreases. The method of magnetization significantly influences the value of the magnetic field. Compared to the field measured for a non-magnetized bar, the use of magnetic excitation in any configuration of the magnets causes an increase in the field value. As a result, the magnetic field diagrams obtained for different cover thicknesses *h* differ significantly, which facilitates identification. The strongest field over the rebars was measured in the case of magnets directed towards the bar with homonymous poles (SPM), lower for magnets with opposite poles (OPM), and the lowest for the reference sample in which the rebar was not magnetized. One can observe that the maximum value of the magnetic field component *B_z_* was similar in both magnetization methods.

In the case of non-magnetized rebar, the graphs representing the magnetic field along the *x*-axis perpendicular to the rebar did not change significantly with increasing cover thickness. Even the changes measured for thickness *h* above 50 mm are minimal.

**Figure 17:**
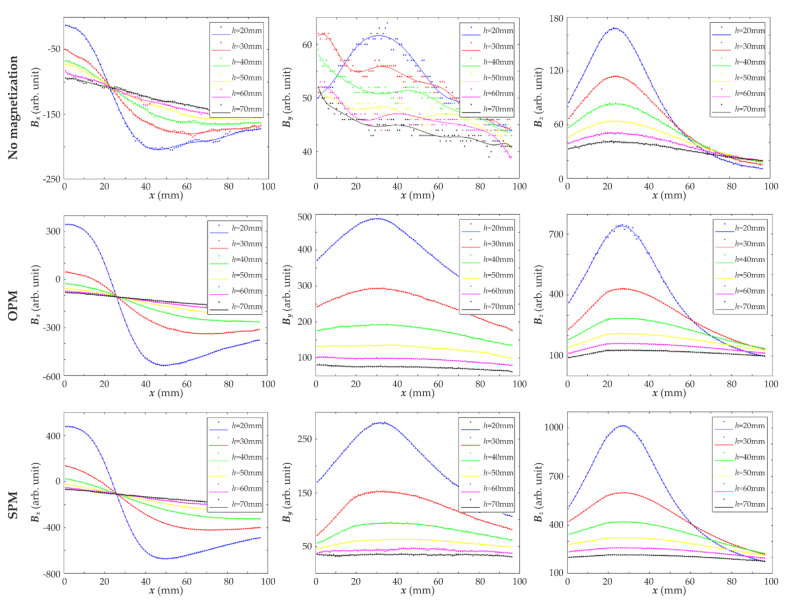
Plots of the magnetic field components as a function of sensor position *x* (mm) which are showing the influence of the concrete cover thickness *h*.

The results obtained with the SPM magnetization system are the easiest to interpret. The *B_x_* component of the magnetic field is particularly interesting. It has a much larger value than the others and changes significantly with increasing cover thickness. Moreover, in contrast to magnetization OPM, the SPM looks similar, regardless of the measurement place in the *y* axis direction. The most important conclusion from the presented results is that the cover thickness *h* can be estimated based on the slope of the graph of the measured magnetic field (Figure 17).

The measurements show that the lack of magnetization causes a significant reduction of the magnetic field and, therefore, it may cause errors in the rebars identification. For example, in Figure 17, in the case of non-magnetized rebar, the *B_y_* component takes very small values. The results of experiments show that the magnetization method can impact noise immunity. Signal to noise ratio calculated for different methods of magnetization and different thicknesses of concrete cover *h* is provided in Table 2.

**Figure 18:**
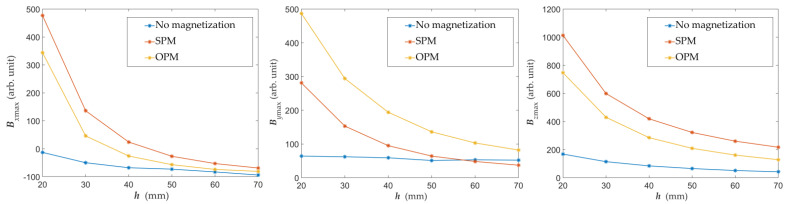
Graphs of the maximum value of the magnetic field components as a function of the concrete cover thickness *h* obtained for various magnetization methods.

As mentioned, the signal to noise ratio (SNR) for *y*-component and non-magnetized rebar has very small values. Moreover, the presence of rebar nearby does not appear to be the dominant factor that formed this characteristic (due to the influence of external fields). Therefore, SNR is not calculated in that case. The impact of noise is much higher in the case of non-magnetized rebars. A slightly bigger SNR was achieved for the SPM magnetization compared to the OPM. However, in this respect, both methods are comparable. As it is not difficult to predict, the growth of the thickness of concrete cover has a negative effect on the SNR. The influence of *h* on the SNR is different for different magnetization methods and for different components. However, drawing conclusions based on Table 2 could be premature due to a small test attempt. In addition, in all cases, the impact of noise is moderate. It can be noted that the dominant influence on SNR has the maximum value of the obtained signal. The relationship between the signal value received from the AMR sensor and the thickness of the concrete cover *h* for different magnetization methods is shown in Figure 18.

**Figure 19:**
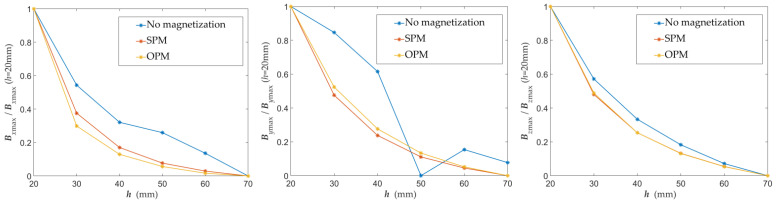
Graphs of the normalized maximum values of the magnetic field components as a function of the concrete cover thickness *h* obtained for various magnetization methods.

The maximal value of signals presented in Figure 18 is greater in the case of the *B_x_* and *B_z_* for SPM (same pole magnetization). In the case of *B_y_*, the biggest signal value is achieved for OPM (opposite pole magnetization). One can observe that the maximal signals are significantly smaller without magnetic excitation. Results of identification, in that case, are uncertain (nevertheless, detection of the reinforcement is possible). The problem of measurements conducted without magnetization is the low value of the received signals. This problem causes strong noise influence and the characteristics ambiguity. It is worth noting that the maximum amplitude of various components is significantly different. The maximal value of the signal obtained for *B_z_* is much higher than for the two other components. This fact has a substantial impact on the SNR. The comparison of the characteristics for the non-magnetized rebars, magnetized with the SPM and magnetized with the OPM, is shown in Figure 19 (normalized curves). For non-magnetized rebars, there are differences in the shape of the characteristics caused by noise and the ambiguous polarity of the rebars. As a result, they are challenging to interpret, and the identification results could be inaccurate. Differences between maximum values of signals obtained for SPM and OPM are minor. Obtained results in these two cases are comparable.

**Figure 20:**
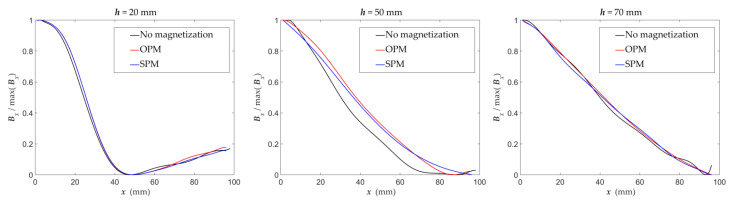
Graphs of the normalized value of the magnetic field component *B_x_* (*x*) as a function of the concrete cover thickness *h* (mm) obtained for various magnetization methods.

The type of magnetization method does not affect the steepness of changes in the measured linear profiles of the magnetic field components (Figure 20). However, also in this aspect, low SNR makes identification difficult in the case of lack of magnetization. The curves obtained for SPM and OPM are almost the same.

**Figure 21:**
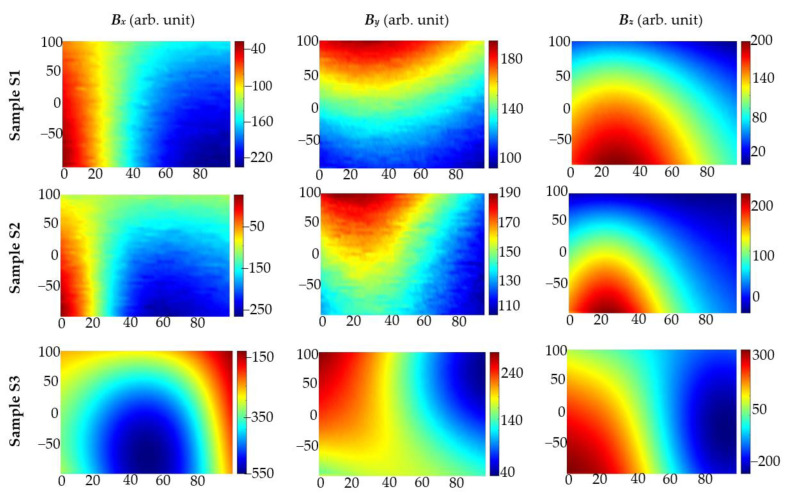
Results of 2D measurements using AMR sensor obtained for different samples (S1, S2, S3); without magnetization; thickness of the concrete cover *h* = 40 mm; *x* (mm), *y* (mm)—sensor positions.

The following experiment was carried out to investigate the influence of magnetization on identifying the reinforcement mesh. Two kinds of specimens were tested: sample S2—(two rebars one over the other—Figure 15b) and sample S3 (three rebars are next to each other—Figure 15c) are considered in the tests and compared with measured earlier sample S1. The results are presented in Figure 21.

The obtained results indicate that regardless of the method of magnetization or the lack of it, more complex structures containing several bars next to each other (samples S2 and S3) generate field distributions significantly different than in the case of a single bar (sample S1). Correctly-configured magnetic excitation creates opportunities to correctly identify complex structures, which are more similar to existing building structures.

**Figure 22:**
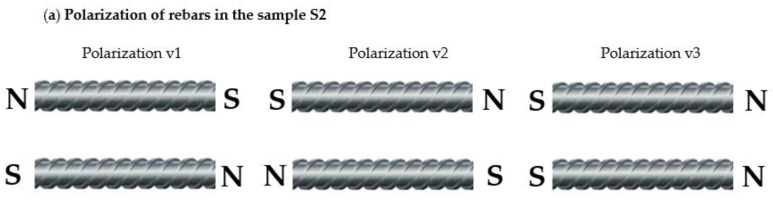

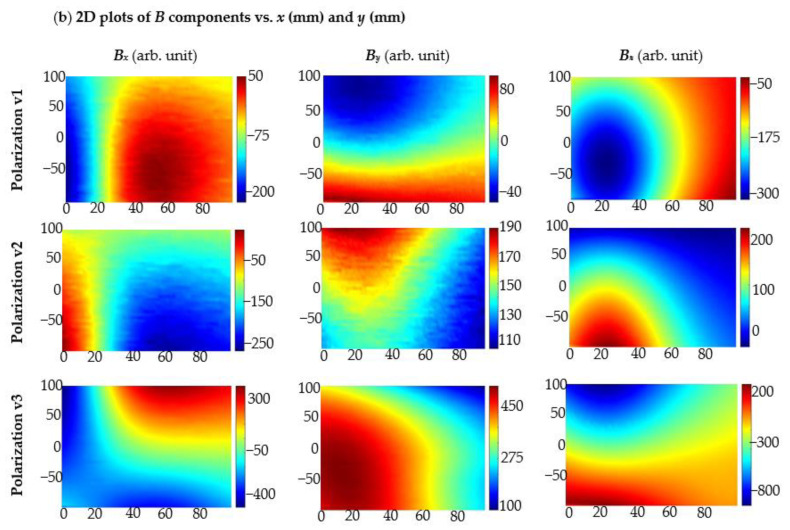
(**a**) Polarizations (residual magnetization) of earlier magnetized rebars in the sample S2; (**b**) Results of 2D measurements using AMR sensor received for different arrangements of earlier magnetized rebars; without external magnetization during measurements, the thickness of the concrete cover *h* = 40 mm; sample S2; *x* (mm), *y* (mm)—sensor positions.

There are many problems with testing reinforcement meshes, where more than one rebar strongly influences the sensor. In the case of concrete structures without magnetization (RMT) the most significant problem is a lack of knowledge about the residual magnetization of individual rebars. Another obstacle is that the rebars could be strongly magnetized during earlier operations (e.g., by a crane with an electromagnetic gripper) and the obtained results strongly depend on the magnetized rebars relative position as shown in Figure 22.

The problem with unknown residual magnetization disappears when magnetic excitation is used. Moreover, the signal value is higher and the identification process is reliable. Next, experiments were conducted for three different samples with the use of different magnetization methods.

**Figure 23:**
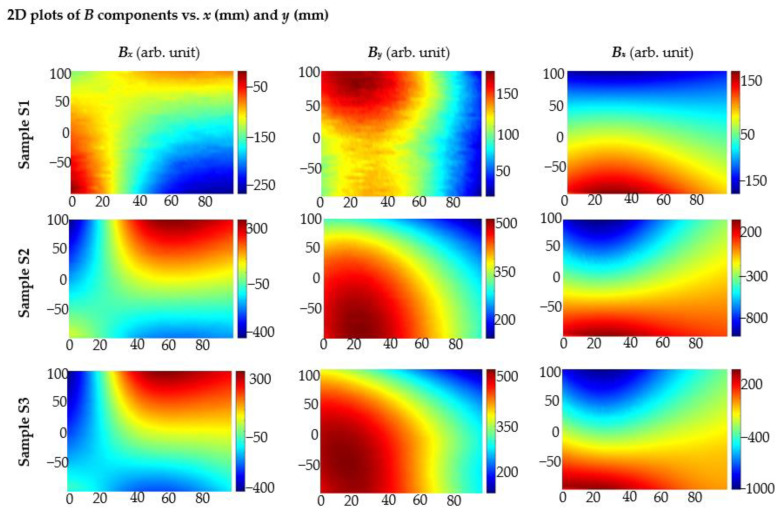
Results of 2D measurements using AMR sensor received for different samples (S1, S2, S3); the magnetization OPM; the thickness of the concrete cover *h* = 40 mm; *x* (mm), *y* (mm)—sensor positions.

In the case of SPM, the value of the obtained signal is bigger than without the magnetization. Unfortunately, identifying the arrangement of the bars in the mesh is very difficult or even impossible. The shapes and maximal values of received characteristics are very similar for sample S2 and sample S3, as shown in Figure 23.

**Figure 24:**
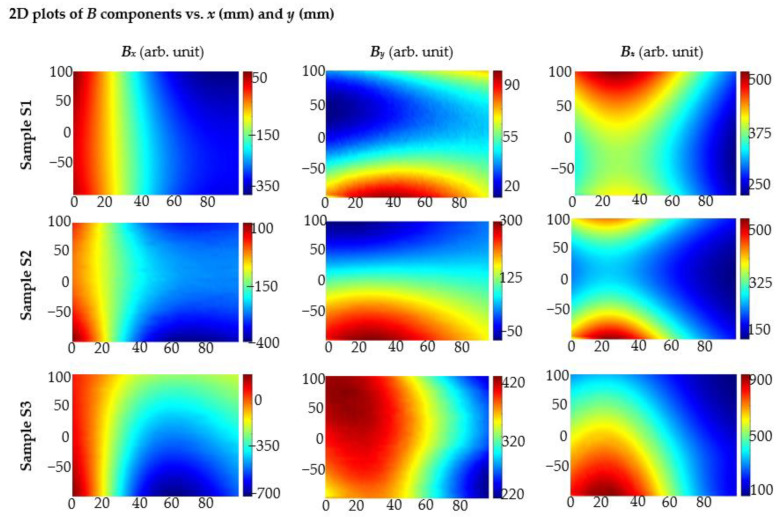
Results of 2D measurements using AMR sensor received for different samples (S1, S2, S3); the magnetization SPM; the thickness of the concrete cover *h* = 40 mm; *x* (mm), *y* (mm)—sensor positions.

Experiments prove that SPM is far superior to OPM in identifying complex structures. For sample S2, in which the bars are located one after the other, the characteristics are to some extent similar to those obtained for single rebar. However, their shapes and maximal values differ enough, and they are easy to distinguish. Therefore, it is possible to easily recognize this arrangement of rebars and even estimate the distance between them. In the case of sample S3, where the rebars are next to each other, the greatest signal values are obtained over the middle rebar (over which the magnets are placed). In addition, this case is easy to recognize. The SPM-results are presented in Figure 24.

## 4. Discussion

The use of magnetic excitation is crucial for the quality of the results in the magnetic evaluation of reinforced concrete structures. In the case of simple structures, where only one rebar is detectable, it affects noise immunity (Table 2) and the signal value. In addition, even a weak magnetic field makes the rebar’s polarization predictable, which significantly facilitates identification. As shown in Figure 19, the results of measurements obtained without magnetization are challenging to predict and heavily dependent on residual magnetization (which can be unknown to the investigator). Generally, the identification of any parameters without magnetic excitation is a subject of significant uncertainty. However, it is possible to detect the rebar even without the magnetization. In the case of more complex structures (Sample S2 and S3), identifying the structure can be tricky when two or more rebars of unknown polarization affect the sensor.

The thickness of the concrete cover (*h*) can be estimated using the magnetic method. The relationship between the signal value and the *h* for different magnetization methods is shown in Figure 18. Potentially also different parameters of a reinforced concrete structure can be tested with this method (e.g., rebars diameter, rebars class, etc.). However, confirmation requires further investigations.

The magnetization method significantly impacts the results of measurements performed with the magnetic method. This aspect is often undervalued. In the case of sample S1, signal value and SNR depend on magnetization methods. Better results are received mostly for SPM (single pole magnetization). Moreover, in the case of the SPM, identification was more straightforward, as the results received for *B*_x_ are similar over the entire surface above the rebar (Figure 15). The magnetization method is even more critical in evaluating more complex structures. In the case of samples S2 and S3, it was possible to identify the structure only by using the SPM (Figure 23 and Figure 24).

The MO sensors enable the evaluation of large areas of reinforced concrete structures in real-time. It is also helpful for fast pilot studies. In the case of greater concrete cover thicknesses, it is necessary to magnetize the rebars due to the moderate sensitivity of the MO-sensor. The signal to noise ratio (SNR) in the case of MO-sensors is much lower than in the case of the AMR sensor. Therefore, for more accurate tests, MO-sensors are not well suited. However, the quality of the results can be improved by hardware enhancement.

The AMR sensors enable effective testing of reinforced concrete structures without magnetization (with typical concrete cover thickness). However, when the concrete cover thickness is high, it is worth using even a small level excitation to improve the system’s efficiency. This solution provides a stronger signal, easier to interpret and analyze the characteristics.

MR elements can be used for area testing. For this purpose, matrices of the sensors can be used. The experiments presented in the paper show that the elements of this kind are much more sensitive and resistant to noise than MO. Comparison is presented in Table 3. The MR sensors also allow the testing of particular spatial components *B_x_*, *B_y_*, *B_z_*. However, these elements cannot be used if the magnetic field is out of range. Therefore, if the magnetic field can be stronger than 1 mT, it is recommended to use a proper MO-sensor or matrix of Hall-elements (Hall-elements possess all advantages of MR sensors, but the active range is much higher than in the case of AMR and the sensitivity is comparable with MO-sensors).

## 5. Conclusions

In the introduction of this paper, it has been shown that only two groups of nondestructive testing (NDT) methods enable direct and effective testing of the condition of reinforced concrete structures. A complete comparison of various NDT methods used in civil engineering is presented in [3]. Magnetic and electromagnetic tests are better suited for reinforcement testing than those that use a mechanical wave. Electromagnetic and magnetic waves affect mainly/only steel bars. Concrete is for such waves (almost) transparent. As shown in [3] magnetic tests can be used for very similar purposes as the eddy current (EC) method. However, the tested method has several significant advantages over electromagnetic evaluation (particularly the EC tests with which they can compete). The experiments show that the most significant advantage is the ability to perform area testing, which would be difficult to do with, e.g., EC tests.

Moreover, the excitation system in magnetic studies does not require advanced power electronic systems or even a power supply. This makes a magnetic method very cheap in implementing and universal in application. The next advantage lies in received data. Research results are relatively simple in interpretation (especially with a well-designed excitation system). Interpretation is even simpler than in the case of EC tests and much simpler than in the case of GPR. The last huge advantage of magnetic testing is the possibility to analyze particular spatial magnetic components, which, combined with the area test, creates unique possibilities which no other method gives.

The magnetic test also has limitations. Compared to EC testing, their spatial resolution is firmly limited. Compared to GPR tests, the effective range is small. Nevertheless, the possibility to performing the area tests in a simple way, cheap and straightforward hardware implementation, simplicity of the interpretation of results, and the ability to test for particular spatial components *B_x_*, *B_y_*, *B_z_*, makes the method universal and useful in the evaluation of composite structures (in particular structures of reinforced concrete).

The results of the experiments presented in this paper prove that AMR sensors are well suited for area tests. The sensor, unlike MO enables the study of particular spatial components *B_x_*, *B_y_*, *B_z_*. They are also more sensitive and more resistant to noise. In addition, they are linear and there is no hysteresis phenomenon. The disadvantage of matrixes of AMR sensors is the relatively small availability on the market. Moreover, in the case of AMR, BGA assembly is required, which makes such transducers challenging to build without proper equipment. However, with professional assembly, such sensors compete with the MO sensors. The MO sensors are not destroyed when a tested magnetic field is too strong, they have a high resolution and in many cases, measurement results do not require any processing. At this moment, the superiority of the MR sensor matrix over MO sensors cannot be clearly stated. Principles of operation of MO and MR sensors are completely different. Moreover, both sensors have some advantages. For example, at low *h* and relatively strong excitation, MO sensors ensure high resolution at relatively high (sufficient) SNR. For the same conditions, the AMR sensor can be damaged due to the too strong magnetic field. Simplifying, AMR matrices transducers are better for testing a weak magnetic field when the MO sensors are better suited to a strong field. Therefore, further comparative studies will be continued. A simple AMR sensor matrix has already been constructed for this purpose.

Experiments have shown that the efficiency of identifying the concrete cover thickness *h* may be in the case of magnetic methods similar to the efficiency of identifying with EC tests (very high for standard concrete cover thicknesses). In further studies, the possibility of identifying diameter and class (alloy from which rebars are made) will also be tested. Identification of such parameters is possible using EC Tests [8,9,10,11]. In the case of the EC system, the frequency and amplitude of the excitation are the main factors determining the efficiency of the method. Similarly, in the case of magnetic methods, the configuration of excitation magnets can be crucial for the identification of reinforced concrete structures. Moreover, component *B_x_*, *B_y_*, *B_z_* analysis can be fundamental for more reliable evaluation.

## Figures and Tables

**Table 1:** Signal to noise ratio SNR calculated for measurements obtained by MO sensor for different concrete cover thicknesses *h*.

*h* (mm)	0.5	20	40	60	80	100
SNR (dB)	29.7	26.9	24.8	23.0	22.5	22.0

**Table 2:** Signal noise ratio SNR (dB) calculated for measurements obtained using AMR sensor for different concrete cover thicknesses *h*, and different magnetization methods.

*h* (mm)		20	30	40	50	60	70
No mag.	*B_x_*	35	31	25	23	21	21
	*B_y_*	25	X	X	X	X	X
	*B_z_*	38	36	33	29	27	27
SPM	*B_x_*	48	45	37	38	35	34
	*B_y_*	52	47	46	43	41	40
	*B_z_*	58	56	56	55	56	53
OPM	*B_x_*	45	44	44	36	31	29
	*B_y_*	49	49	50	47	48	47
	*B_z_*	49	51	51	50	49	47

**Table 3:** Signal to noise ratio SNR calculated for measurements obtained by MO and AMR sensors for different concrete cover thicknesses *h*, and same pole magnetization.

*h* (mm)		20	40	60
*MO*		27	25	23
AMR	*B_x_*	48	37	35
	*B_y_*	52	46	41
	*B_z_*	58	56	56

## Data Availability

The data presented in this study are available on request from the corresponding author. The data are not publicly available due to a complicated structure that requires additional explanations.
